# Radiation Dose-Rate and DNA Damage

**DOI:** 10.1289/ehp.1205595

**Published:** 2012-11-11

**Authors:** Peter Melzer

**Affiliations:** brainmindinst.com, Charlottesville, Virginia, E-mail: melzerpeter@gmail.com

In their article, Olipitz et al. (2012) examined signs of DNA damage after chronic exposure of C57Bl6 mice to low-level ionizing radiation (3 mGy/day). For 5 weeks, mice were irradiated continuously with 35.5 keV X-rays produced by the decay of iodine-125, yielding an accumulated dose of 105 mGy. The observed effects were compared with those from acute irradiation by X-ray machine at 1,700 mGy/day up to the same accumulated dose.

Olipitz et al. (2012) investigated signs of DNA damage using four histological methods. Most prominent of the methods was the expression of functional fluorescent protein as a result of recombination by homology-directed repair in pancreatic cells of transgenic FYDR (fluorescent yellow direct repeat) mice derived from the C57Bl6 strain. The other three methods were carried out using genetically unaltered C57Bl6 mice. The authors investigated DNA base damage in splenocytes; DNA double strand breaks in bone marrow erythrocytes; and the expression of select genes implicated in cell cycle arrest, tumor suppression, and apoptosis in white blood cells from blood samples.

Olipitz et al. (2012) used equal numbers of unirradiated mice as controls. However, sample sizes across the study ranged from 6 to 60 animals. Because of the wide range in animal numbers, nonparametric methods should have been used in statistical analyses. A multivariate analysis of variance comprising all observations in the study should have preceded any pairwise comparisons to allow the authors to evaluate the variability of observations within samples compared with the variability among samples (Mickey and Dunn 2009). Furthermore, the use of transgenic mice with one method and unaltered mice with the other three might have increased the variability in observation, reducing the chance of detecting statistically significant differences. The above weaknesses in experimental design and statistical analysis may have profoundly compromised the authors’ ability to discover statistically significant effects of chronic exposure to low-level ionizing radiation.

In the “Discussion” of their paper, Olipitz et al. (2012) stated that

Chromosome aberrations offer an alternative approach for detecting chromosome breaks, and using this approach, others have shown that low dose-rate radiation indeed induces aberrations *in vitro* (although the dose-rate was approximately 10-fold higher than that used in the present study) (Tanaka et al. 2009).

However, in the paper cited by Olipitz et al., Tanaka et al. (2009) noted that

Significant changes in regression coefficients (b3, b4 and b6) obtained by multiple linear regression analysis, which are the same as a coefficients in the linear regression lines, revealed that dose-rate effects on the incidence of unstable-type aberrations were found at dose rates of 1, 20 and 400 mGy/day.

One milligray per day equals about one-third of the dose-rate Olipitz et al. (2012) used for chronic exposure. Tanaka et al. (2009) irradiated mice with γ-radiation at 1 mGy/day for more than a year to establish a statistically significant dependence of splenocytic chromosomal aberrations on exposure dose. In mice at 438 days of irradiation (494 days of age), Tanaka et al. observed a mean frequency of dicentric chromosomes more than twice as high (0.38 ± 0.15 per 100 cells) as the spontaneous frequency determined in unirradiated mice of similar age (556 days; 0.17 ± 0.14 per 100 cells). Thus, the findings of Tanaka et al. (2009) suggest that Olipitz et al. (2012) might have detected DNA damage if they had exposed the mice to low-level radiation for a longer time.

In addition, roughly half the accumulated dose Olipitz et al. (2012) used may be effective in children. Results of a recent study suggest that patients subjected to CT (computed tomography) scans as children incur a 3-fold greater risk for developing leukemia and brain cancer at accumulated doses of 50 mGy and 60 mGy, respectively (Pearce et al. 2012).

Finally, in an actual radiological emergency, multiple environmental factors may interact synergistically to effect DNA damage. For example, inflammatory responses may stimulate cell division, increasing the likelihood for ionizing radiation to cause DNA strand breaks. Although Olipitz et al. (2012) investigated only the effects of external exposure to ionizing radiation, internal exposure may pose a greater risk to public health in the 50-mile ingestion zone anticipated in U.S. emergency action plans.

## References

[r1] Mickey RM, Dunn OJ (2009). Applied Statistics: Analysis of Variance and Regression.. New York:Wiley and Sons.

[r2] Olipitz W, Wiktor-Brown D, Shuga J, Pang B, McFaline J, Lonkar P (2012). Integrated molecular analysis indicates undetectable change in DNA damage in mice after continuous irradiation at ~ 400-fold natural background radiation.. Environ Health Perspect.

[r3] Pearce MS, Salotti JA, Little MP, McHugh K, Lee C, Kim KP (2012). Radiation exposure from CT scans in childhood and subsequent risk of leukaemia and brain tumours: a retrospective cohort study.. Lancet.

[r4] Tanaka K, Kohda A, Satoh K, Toyokawa T, Ichinohe K, Ohtaki M (2009). Dose-rate effectiveness for unstable-type chromosome aberrations detected in mice after continuous irradiation with low-dose-rate γ rays.. Radiat Res.

